# Validation of an active shape model-based semi-automated segmentation algorithm for the analysis of thigh muscle and adipose tissue cross-sectional areas

**DOI:** 10.1007/s10334-017-0622-3

**Published:** 2017-04-28

**Authors:** Jana Kemnitz, Felix Eckstein, Adam G. Culvenor, Anja Ruhdorfer, Torben Dannhauer, Susanne Ring-Dimitriou, Alexandra M. Sänger, Wolfgang Wirth

**Affiliations:** 1 Institute of Anatomy, Paracelsus Medical University, Strubergasse 21, 5020 Salzburg and Nuremberg, Austria; 2 Chondrometrics GmbH, Ainring, Germany; 3 La Trobe Sports and Exercise Medicine Research Centre, La Trobe University, School of Allied Health, Bundoora, Australia; 4 Paris Lodron University, Salzburg, Austria

**Keywords:** Segmentation, Statistical shape model, Thigh muscle, Training intervention, Magnetic resonance imaging

## Abstract

**Objective:**

To validate a semi-automated method for thigh muscle and adipose tissue cross-sectional area (CSA) segmentation from MRI.

**Materials and methods:**

An active shape model (ASM) was trained using 113 MRI CSAs from the Osteoarthritis Initiative (OAI) and combined with an active contour model and thresholding-based post-processing steps. This method was applied to 20 other MRIs from the OAI and to baseline and follow-up MRIs from a 12-week lower-limb strengthening or endurance training intervention (*n* = 35 females). The agreement of semi-automated vs. previous manual segmentation was assessed using the Dice similarity coefficient and Bland-Altman analyses. Longitudinal changes observed in the training intervention were compared between semi-automated and manual segmentations.

**Results:**

High agreement was observed between manual and semi-automated segmentations for subcutaneous fat, quadriceps and hamstring CSAs. With strength training, both the semi-automated and manual segmentation method detected a significant reduction in adipose tissue CSA and a significant gain in quadriceps, hamstring and adductor CSAs. With endurance training, a significant reduction in adipose tissue CSAs was observed with both methods.

**Conclusion:**

The semi-automated approach showed high agreement with manual segmentation of thigh muscle and adipose tissue CSAs and showed longitudinal training effects similar to that observed using manual segmentation.

**Electronic supplementary material:**

The online version of this article (doi:10.1007/s10334-017-0622-3) contains supplementary material, which is available to authorized users.

## Introduction

Deficits in thigh muscle strength are known to be associated with knee pain and functional limitations [1, 2] and to increase the risk of both incident knee osteoarthritis (OA) [3–5] and knee replacement surgery [6]. Thigh muscle weakness can be caused by diseases such as sarcopenia [7] or chronic obstructive pulmonary disease [8], but thigh muscle strength also declines physiologically as part of the normal aging process [7]. Measurements of muscle strength are clinically useful, but depend on the willingness of the study participants to exert maximal possible effort, and also are potentially biased by the presence of pain [9].

Magnetic resonance imaging (MRI) permits visualization of thigh muscle cross-sectional areas (CSA) directly, with CSAs being correlated with clinical measures of muscle strength [10, 11]. Yet, a comparative study has shown that thigh muscle CSAs are more sensitive to longitudinal change in knee OA than isometric muscle strength measures [12]. Further, MRI is able to directly depict thigh adipose tissue. It is therefore able to detect subtle variations in both muscle morphometry and thigh tissue composition [13], which has been suggested to be of relevance for knee function [14, 15]. MRI is therefore increasingly used to study the association between thigh tissue composition and knee OA [11, 13, 16–18] and to investigate the impact of training interventions on thigh composition [19, 20] as well as on functional and clinical outcomes of knee OA. However, morphometric analyses of thigh tissues require image segmentation, and the time needed for manual thigh CSA segmentations precludes the analysis of large databases and image repositories, such as the Osteoarthritis Initiative (OAI) [21]. Previously, several groups [22–29] have presented software solutions for automated thigh volume and CSA segmentation and have pursued different approaches to overcome the challenges in capturing the complex morphology of thigh muscle and adipose tissue, which is complicated by considerable inter-subject variability. While these methods showed potential for the use in cross-sectional studies, importantly, they were not validated to assess longitudinal changes in thigh tissue composition. With large longitudinal MRI repositories of several thousand patients currently being available for public use (e.g., the OAI), fast and reliable semi-automated segmentation methods are required that can capture longitudinal change in thigh composition with a reasonable sensitivity to change.

The objective of the current study was therefore to: (1) determine the agreement between a semi-automated thigh muscle and adipose tissue segmentation method based on an active shape model (ASM) combined with an active contour model (ACM) post processing vs. manual segmentations and (2) to validate the semi-automated image analysis technique for the longitudinal analysis of change in thigh muscle and adipose tissue CSAs vs. previously reported changes observed using manual segmentations [19]. The latter was deemed of particular importance as one of the potential advantages of the application of the image algorithm to large longitudinal image repositories is its ability to detect small changes over time that may not be detected by clinical strength measurements [12].

## Materials and methods

### Semi-automated segmentation method

**Fig. 1:**
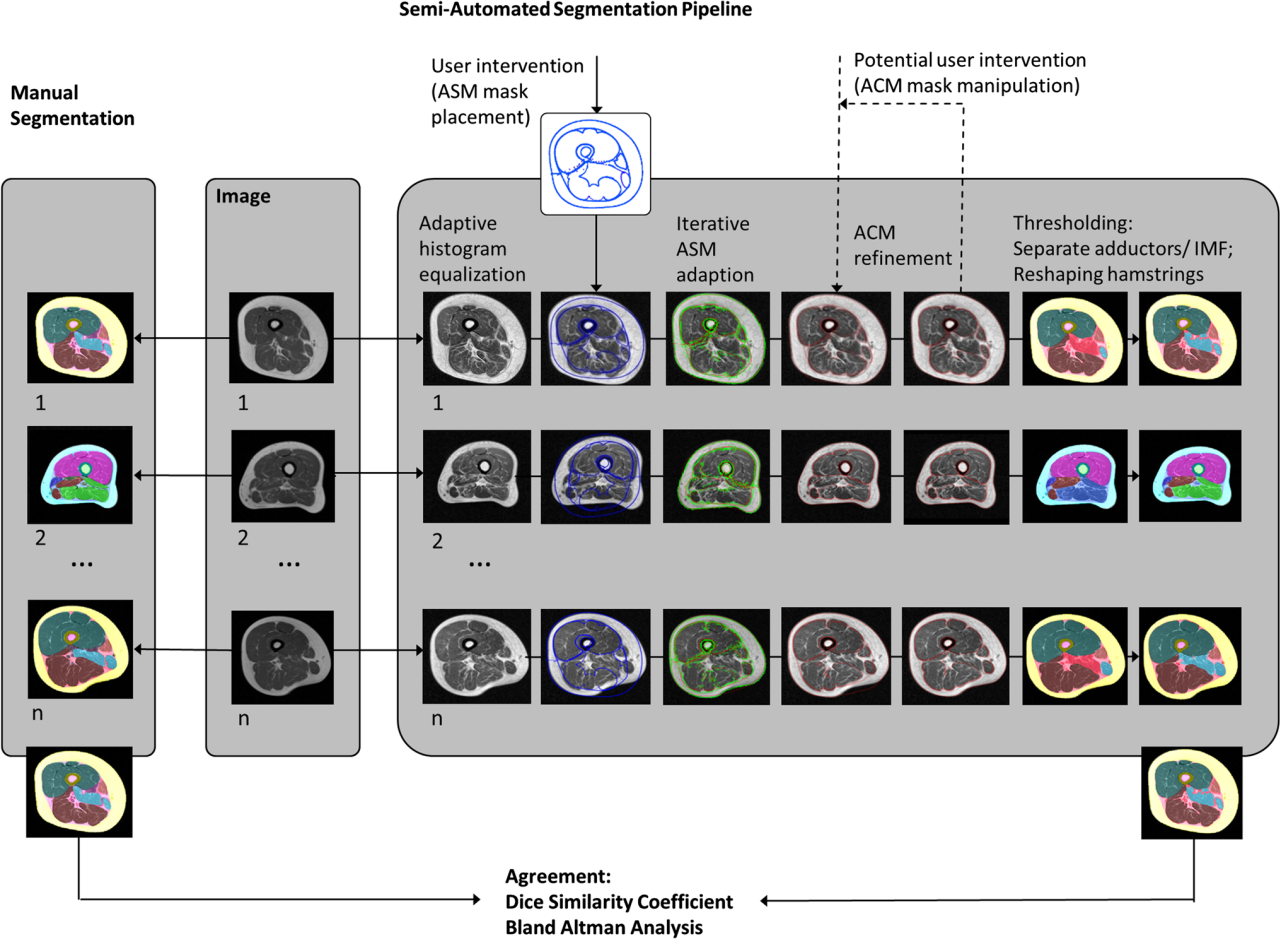
Comparison of manual and semi-automated segmentation of thigh MR images; semi-automated segmentation pipeline: adaptive histogram equalization; manual placement of active shape model (ASM) mask on the thigh; locating individual thigh shapes by iterative ASM adaption; refining individual thigh structures using active contour models (ACM);* dashed lines* for potential user intervention; application of thresholding to separate the adductors and IMF (not part of ASM/ACM); reshaping the hamstrings; subsequent comparison of the agreement between manual and semi-automated segmentation using the dice similarity coefficient (DSC) and Bland-Altman analyses

**Fig. 2:**
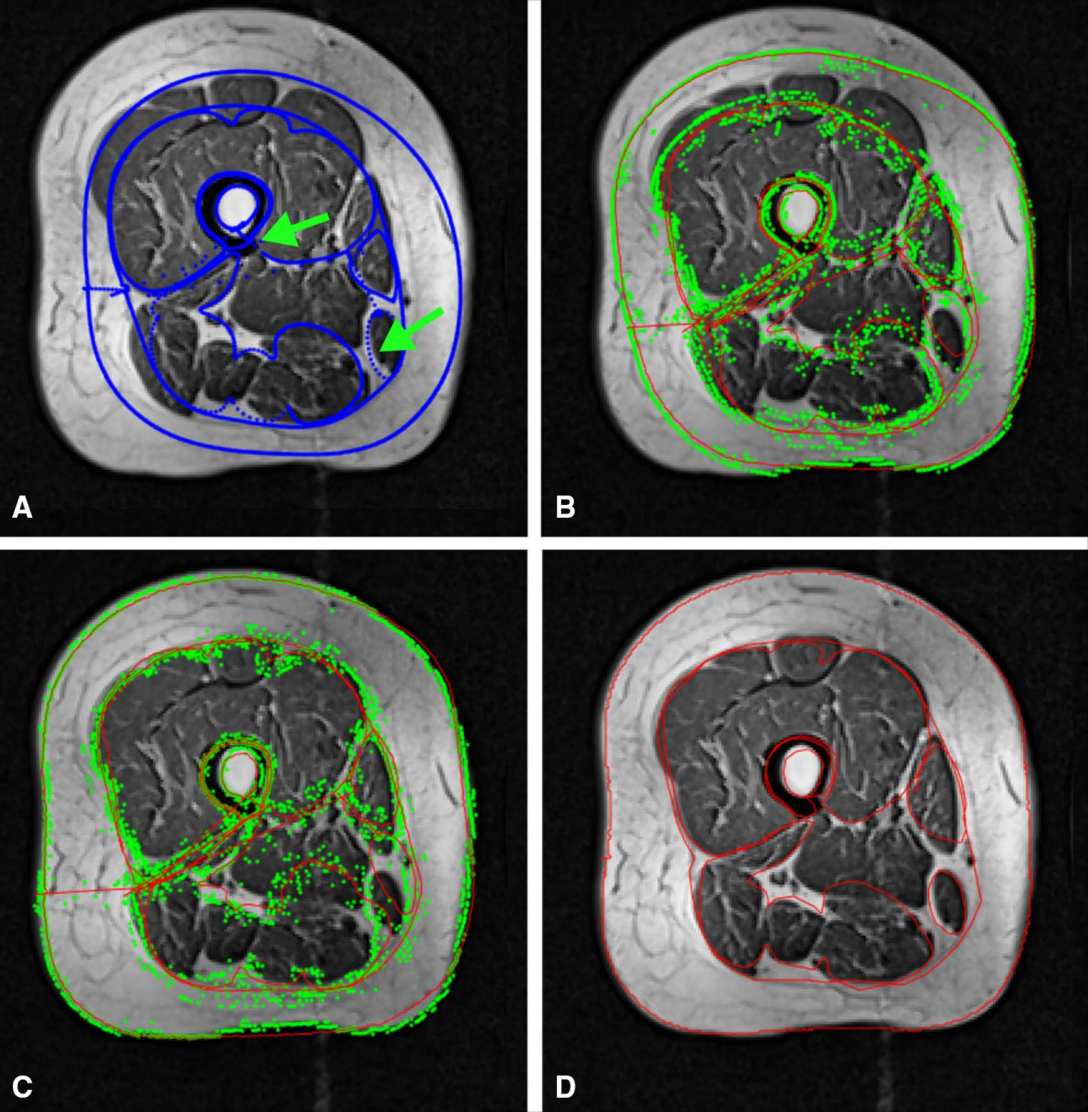
The active shape model (ASM) iteratively adapts to the shape of the thigh cross-sectional areas: **a** initialized ASM mask on target image aligned with the femoral bone and the gracilis muscle; **b** ASM mask after three iterations; **c** ASM mask after 20 iterations; **d** results of the ASM model after 30 iterations

Semi-automated segmentation of axial MR images of the thigh was implemented using a graphical user interface application in MATLAB (version R2015a, Natick, MA, USA). In a first step, the image contrast was enhanced using adaptive histogram equalization (Fig. 1), and the image was subsequently filtered by a 2 × 2 median filter before the ASM (“Active Shape Model and Active Appearance Model” library, Kroon) [30] mask was manually placed on the axial MR images using the femoral bone and the gracilis muscle position as reference (Fig. 2). The ASM iteratively adapts the model shape to the image (Fig. 2), with the model shape being flexible and able to deform within the “shape variation set” as guided by a training data set [31]. The user could adapt the size and rotation angle of the ASM mask to fit the individual thigh shape and rotation, with the mask then being iteratively (*n* = 30 iterations) adapted to the thigh shapes of the target image (Fig. 2). The mask was subsequently divided into several masks, representing the following individual thigh components: thigh circumference, muscle hull (fascia), femoral bone circumference, femoral medulla, quadriceps, hamstrings, sartorius and adductors (gracilis) (Fig. 1).

Because of the large inter-subject variability of these structures, the model-based ASM was not always able to detect the edges of all structures with a sufficiently high accuracy; the user therefore could manually modify the size, position and rotation angle of the individual thigh component masks before the ACMs were applied as a first refinement step (Fig. 1). The ACM (Snakes: Active Contour Models Library, Kumar) [32] is based on a spline minimizing an energy function, which is constrained by internal forces (because of the bending), imaging forces and constraint energy forces [33]. The elasticity (*α*) and rigidity (*β*) controlling the internal spline energy were set to *α* = 0.4 and *β* = 10.0 for the thigh circumference, femur, medulla, muscle hull, quadriceps, gracilis and sartorius and to *α* = 0.9 and *β* = 0.5 for the hamstrings to account for the differences in curvature. The ACMs for refining segmentations of the thigh circumference, muscle hull, quadriceps and hamstrings were applied directly to the unfiltered image, whereas the ACMs for refining segmentations of the medulla and femoral bone circumference were first applied to a 6 × 6 median filtered image before they were refined using unfiltered images. The user could repeat the manual interaction (size, position and rotation angle) multiple times (if needed) before repeating the ACM segmentation step (needed in approximately 50% of the MRIs) (Fig. 1).

**Fig. 3:**
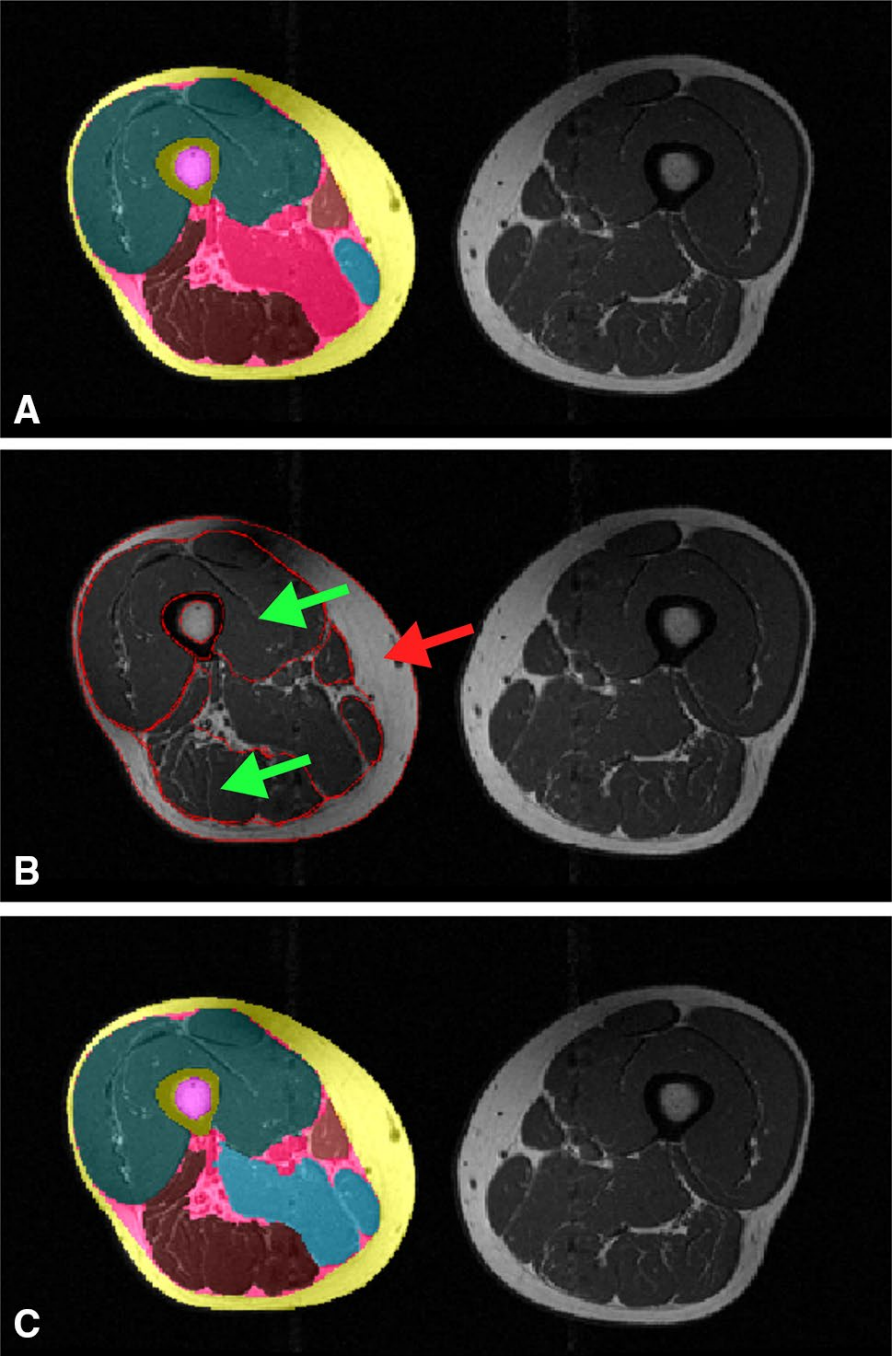
Application of the thresholding step to distinguish between the IMF and adductors; **a** post ASM and ACMs segmentation results; **b** average signal intensity of quadriceps and hamstrings (*green arrow*) and average signal intensity of SCF (*red arrow*) used for distinction between the adductors and IMF by applying a thresholding on the remaining not assigned pixels; **c** final post-thresholding segmentation results

The subcutaneous fat (SCF) segmentation was defined as the difference between the convex hull of the muscle compartment and the thigh circumference. Because not all parts of the adductors were part of the ASM (see Fig. 2), a threshold-based segmentation step based on the mean signal intensity of the SCF $\overline{{{\text{SI}}_{\text{SCF}} }}$ and the mean signal intensity of the quadriceps and the hamstrings ($\overline{{{\text{SI}}_{{{\text{Q}},{\text{H}}}} }}$) was applied to assign the remaining pixels to the IMF (intermuscular fat), the adductors or other intermuscular tissue. The threshold $t = \frac{{\overline{{{\text{SI}}_{\text{SCF}} }} + \overline{{{\text{SI}}_{\text{QH}} }} }}{{2\left( {{\text{pixel}}_{\text{highest}} - {\text{pixel}}_{\text{lowest}} } \right)}}$ was calculated, and pixels with signal intensity < *t* were assigned to IMF, while pixels with ≥ *t* were assigned to adductors if more than 70 pixels were connected to each other or to other intermuscular tissue (veins, etc.) in case of smaller connected pixel groups (Fig. 3).

No manual correction or central quality control of the semi-automated segmentation results was performed because the purpose of this study was to evaluate the “unbiased” semi-automated segmentation method.

### Training of the active shape model

The ASM was trained using 113 axial MR images from the OAI (male: *n* = 49, age 62.3 ± 10.4 years, BMI 28.8 ± 3.4 kg/m^2^; female: *n* = 64, age 59.6 ± 9.3 years, BMI 28.8 ± 4.6 kg/m^2^) in which the muscles, adipose tissue and femoral bone had been manually segmented previously to study the impact of pain [17, 18] and radiographic disease stage [34] on thigh muscle CSAs and the association between thigh muscle CSAs and incident radiographic knee OA [35]. The MR images were acquired with a T1-weighted spin echo MRI sequence from the OAI (slice thickness 5 mm; in-plane resolution 0.98 mm; no inter-slice gap, repetition time 500 ms, echo time 10 ms) [36, 37] using a 3-T scanner (Siemens Trio, Siemens AG, Erlangen, Germany). The segmentation was performed at a slice located at 33% of the femur (from distal to proximal) because this was the anatomical location that was consistently covered by the image acquisition that encompassed 15 slices at a fixed distance from the distal femoral metaphysis [12]. The ASM consisted of 21 anatomical landmarks that were manually marked in the training images and 3562 intermediate points to describe the shapes of thigh structures. Because the adductor longus and magnus muscles were frequently absent in the MRI slices located at 33% of the distal to proximal femur length, only the gracilis muscle of the adductors was included in the ASM.

### Application of the semi-automated algorithm to the OAI and training intervention images

The segmentation method described above was applied to 20 thigh MR images from OAI participants (male: *n* = 6, age 66 ± 9 years, BMI 30.7 ± 4.6 kg/m^2^; female: *n* = 14, age 61 ± 9 years, BMI 29.6.1 ± 5.9 kg/m^2^) who had been previously selected and segmented manually [35]. The MR images were acquired and segmented using the same MRI protocol and manual segmentation method as the training data set and that were not included in the training data set described above. To explore whether the segmentation method can also be applied to thigh MR images acquired on a different MRI scanner, we analyzed MR images from 35 women participating in a 12-week training intervention study [38] that were acquired using an axial T1-weighted spin echo protocol (slice thickness 10 mm, in-plane resolution 0.78 mm, no inter-slice gap, repetition time 1541 ms, echo time 15 ms) on a 1.5-T MRI scanner (NT Interna, Phillips Medical Systems, Best, The Netherlands). In this study, 35 perimenopausal, untrained women with sedentary occupation activities [38, 39] were randomized to 12 weeks of supervised lower-limb strengthening training with varying muscle loading between agonist and antagonist muscles (3 × 60 min per week, *n* = 16, age 51 ± 3 years, BMI 26.1 ± 3.3 kg/m^2^) or to 12 weeks of supervised cycling endurance training (3 × 60 min per week, *n* = 19, age 52 ± 3 years, BMI 26.8 ± 5.8 kg/m^2^). Details of the training protocol have been previously published [38]. The semi-automated segmentation method was applied by a PhD student with blinding to the image acquisition order (baseline vs. follow-up), but not to the training intervention type. The thigh muscle and adipose tissue CSAs were determined from the baseline and 12-week follow-up images of the dominant leg at the same anatomical location at which the algorithm had been trained (i.e., image slice located at 33% of the femur from distal to proximal).

The average segmentation time for the semi-automated approach was 3 min 4 s (range 1 min 31 s–5 min 35 s) for a trained reader and 5 min 30 s (range 2 min 22 s–8 min 16 s) for an untrained reader, depending on the image quality. The computation time was approximately 50 s, which consisted of: (1) ASM computation time of 22 s; (2) ACM computation time of 28 s; (3) thresholding time of ≤1 s. The remaining time was needed for the user interaction including loading and saving the data record. The previously applied manual segmentation approach of the same structures required 60–90 min including quality control by an experienced reader and subsequent corrections [13].

### Active shape model: the cross-validation and minimally required number of training data sets

A twofold cross-validation was used to explore the impact of the specific composition of the training data set on the performance of the ASM. For this purpose, the 113 available training data sets were randomly partitioned into two distinct subsets. The ASM was trained first using the first subset and tested on the second subset, and then the order was switched with the ASM being trained using the second subset and tested on the first subset. This cross-validation step was performed ten times in total (20 applications).

To also explore the minimally required number of training data sets for the ASM, the number of training data sets used for building the ASMs was iteratively increased in steps of *n* = 5, with the resulting ASM models being applied to the *n* = 20 test data from the OAI, for which manual segmentations were available. This analysis was repeated five times for each size of a training data set using different randomizations. Please note that these cross-validation steps were performed without ACM refinement.

### Statistical analysis

All statistical analyses were performed using SPSS version 22 (IBM Corp., USA). To determine the agreement between the manual (M) and the semi-automated (S) technique, the semi-automated and manual segmentation results were compared using the DSC = (2|M ∩ S|/|M| + |S|) [mean ± SD (min–max)] (MatlabR2015a). Additionally, the differences between the manual and the semi-automated results of the training study were examined using Bland-Altman analyses.

Mean and standard deviation observed in the two training intervention groups were reported for baseline values (in cm^2^) as well as for the longitudinal change (in %). Paired *t* tests were used to determine whether significant changes were observed in the two training intervention groups with the semi-automated and/or manual segmentation method and to determine whether the changes measured using the semi-automated segmentation method differed from the changes measured using manual segmentations. The standardized response mean (SRM = mean/SD of the change) was used to measure the sensitivity to change.

The inter-observer variability was assessed by an experienced reader and by a reader without previous experience in segmentation, who underwent formal training with the semi-automated software on three right MRIs of the same cohort.

No central quality control was performed.

## Results

### Active shape model: cross-validation and minimally required number of training data sets

The results from the twofold cross-validation were highly consistent between the 20 experiments (ten samples, with two subsets each—Supplemental Table 1). The DSCs (mean ± SD) were 0.90 ± 0.05 to 0.92 ± 0.05 for the SCF, 0.93 ± 0.03 to 0.94 ± 0.02 for the quadriceps, 0.87 ± 0.05 to 0.89 ± 0.04 for the hamstrings, 0.74 ± 0.13 to 0.80 ± 0.11 for the sartorius, 0.91 ± 0.04 to 0.93 ± 02 for the femoral bone and 0.85 ± 0.06 to 0.88 ± 0.05 for the femoral medulla.

**Fig. 4:**
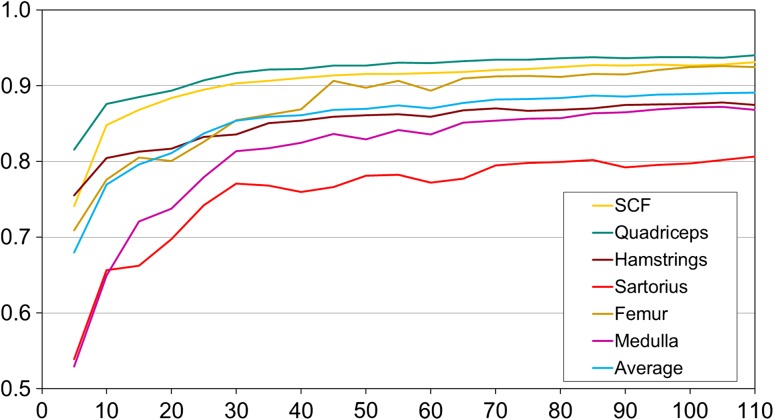
Average Dice similarity coefficients (DSC; agreement between manual and semi-automated segmentation; mean ± SD) obtained from active shape models (ASMs) that were trained using an increasing number of randomly selected training data (in steps of *n* = 5) and were then applied to 20 MRI CSA images from OAI participants to evaluate the segmentation performance

**Fig. 5:**
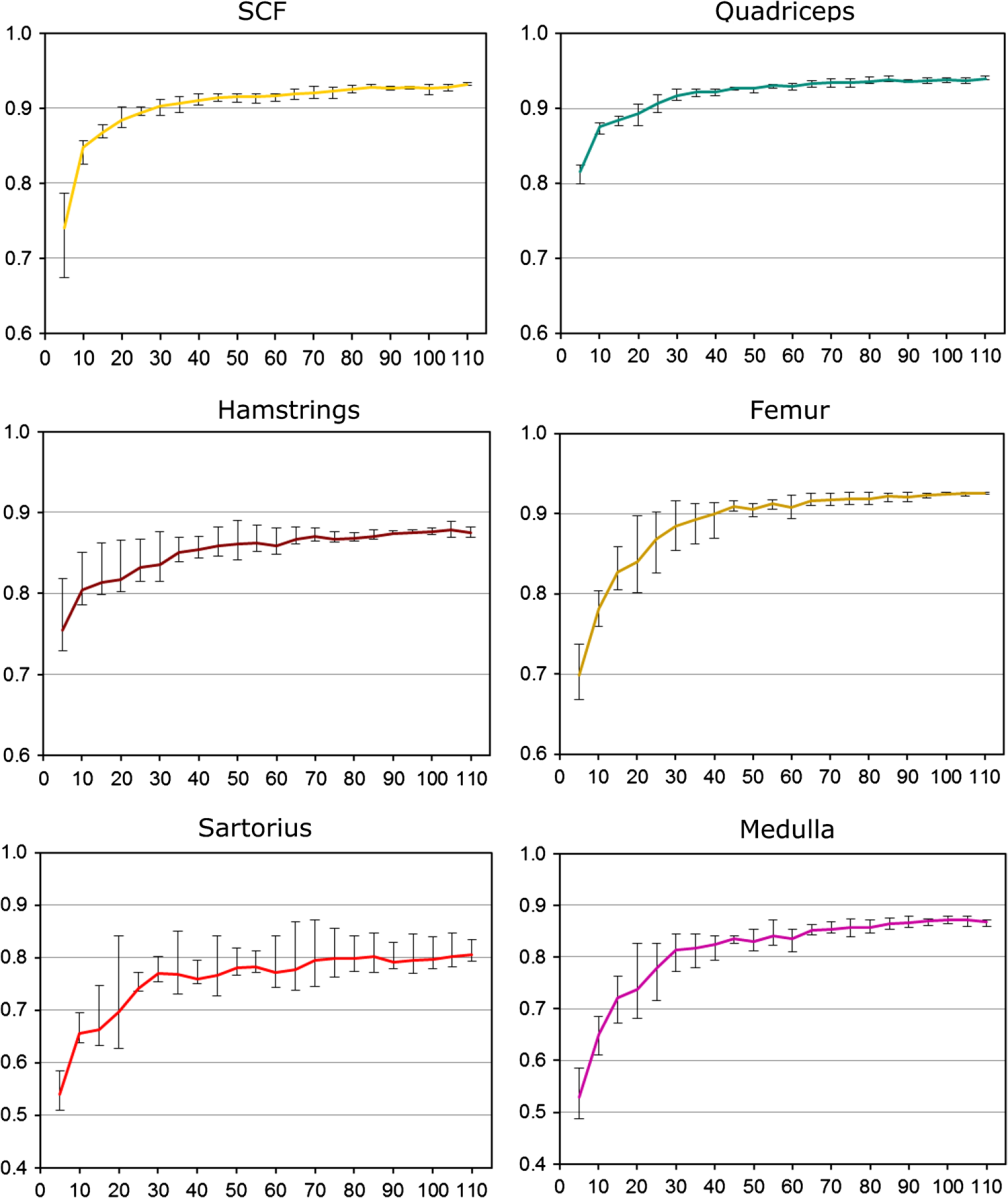
Average Dice similarity coefficients (DSC; agreement between manual and semi-automated segmentation) obtained from the active shape models (ASMs) that were trained using an increasing number of randomly selected training data (in steps of *n* = 5) and that were then applied to 20 MRI CSA images from OAI participants to evaluate the segmentation performance. This analysis was performed five times each using a different randomization, and the *error bars* indicate the minimal and maximal average DSC that was observed for the five runs of the five different randomly training data sets with similar *n*. Please note that the scale differs between graphs

As expected, the performance of the ASM segmentation strongly depended on the size of the training data set, with the models requiring between *n* = 40 and 50 training data sets to achieve satisfactory DSCs. Once this threshold had been exceeded (Figs. 4, 5), further increasing the size only led to small improvements of the ASM segmentation performance (Figs. 4, 5).

### Cross-sectional comparison of automated and manual segmentation results

**Fig. 6:**
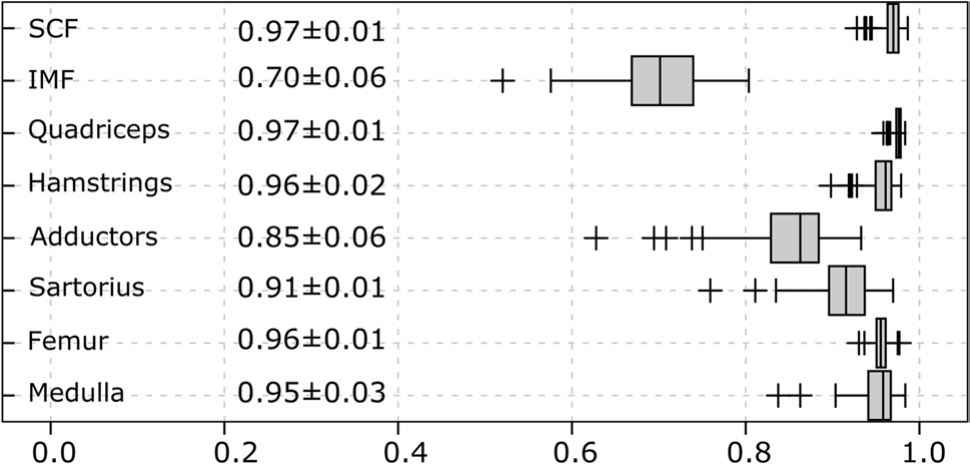
*Box plot* showing the average Dice similarity coefficients (DSC; agreement between manual and semi-automated segmentation; mean ± SD) in 90 MRI CSA images (OAI participants: *n* = 20, training study baseline: *n* = 35 and training study follow-up for *n* = 35 participants). The overall mean DSC over all structures was 0.91 ± 0.09

The DSC (mean ± SD) agreement between semi-automated and manual segmentation was high for the SCF (0.97 ± 0.01), quadriceps (0.97 ± 0.01), hamstrings (0.96 ± 0.02), total femoral circumference (0.96 ± 0.01) and femoral medulla (0.95 ± 0.03) CSAs and was somewhat lower for the adductor (0.86 ± 0.06), sartorius (0.91 ± 0.04) and IMF (0.70 ± 0.06) CSAs (Fig. 6). The DSCs between both methods were very similar for the OAI and training intervention study images (Table 1). In comparison, the DSC was slightly lower and had a larger SD when the ACMs were run without any user interaction to optimize the position of the ACM masks: (DSC SCF 0.94 ± 0.05; quadriceps 0.94 ± 0.01, hamstrings 0.88 ± 0.05, total femoral circumference 0.97 ± 0.97, medulla 0.93 ± 0.01; sartorius 0.82 ± 0.12). The user interaction led to a greater degree of accuracy at the expense of approximately 2 min of time (for an experienced reader).

**Fig. 7:**
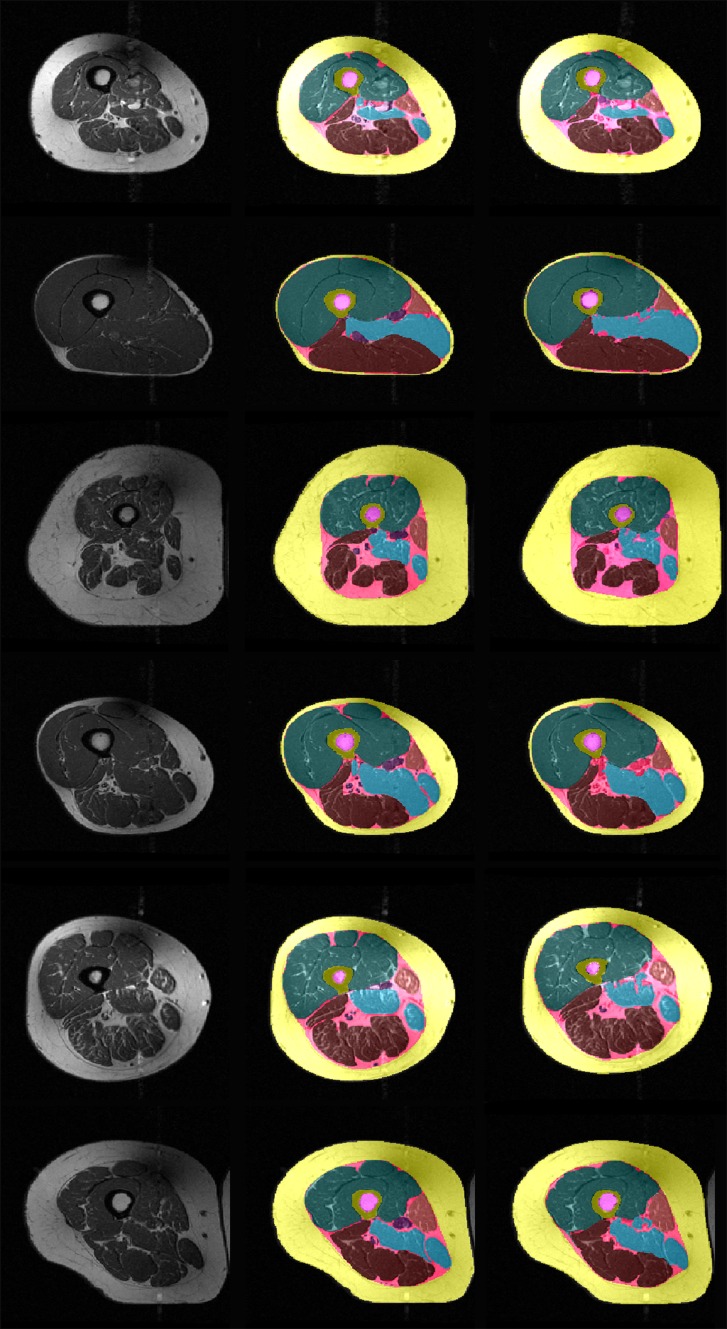
Thigh MRI segmentation results from six OAI participants: *left* original image; *middle* manual segmentation results (quality controlled by an expert reader); *right* semi-automated segmentation results (not quality controlled by an expert reader)

Example segmentation results of the semi-automated method without subsequent quality control by an expert reader and of the manual segmentation method with subsequent quality control by an expert reader are shown in Fig. 7.

**Table 1:** Dice similarity coefficients (agreement between manual and semi-automated segmentation) with average Dice values (mean ± SD) in 90 MRI CSA images (OAI participants, training study baseline and training study follow-up)

	OAI participants (*N* = 20)	Training study (BL) (*N* = 35)	Training study (FU) (*N* = 35)
SCF	0.96 ± 0.01	0.97 ± 0.01	0.97 ± 0.01
IMF	0.72 ± 0.05	0.71 ± 0.06	0.69 ± 0.07
Quadriceps	0.97 ± 0.01	0.97 ± 0.01	0.98 ± 0.01
Hamstrings	0.96 ± 0.01	0.96 ± 0.02	0.96 ± 0.01
Adductors	0.89 ± 0.04	0.85 ± 0.04	0.89 ± 0.07
Sartorius	0.92 ± 0.03	0.91 ± 0.04	0.92 ± 0.03
Femur	0.96 ± 0.01	0.96 ± 0.01	0.96 ± 0.01
Medulla	0.95 ± 0.02	0.95 ± 0.03	0.95 ± 0.03

*OAI* osteoarthritis initiative, *BL* baseline, *FU* follow-up, *SCF* subcutaneous fat, *IMF* intermuscular fat

**Fig. 8:**
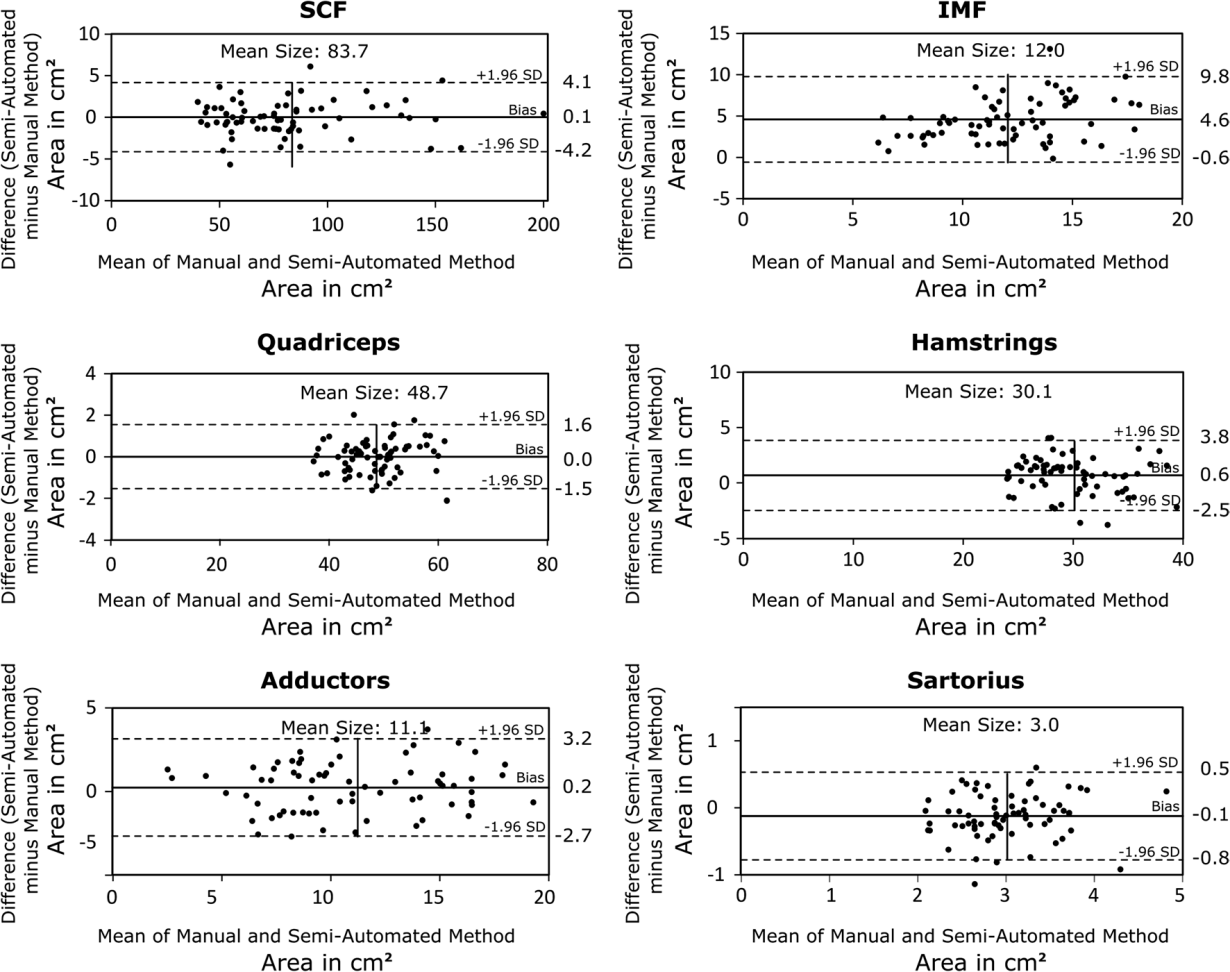
Bland-Altman plots showing the agreement between the manual and the semi-automated segmentation results from the training intervention study. The limit of agreement (±1.96 SD) is shown using *dashed lines*

The Bland-Altman analysis showed good agreement between the semi-automated and manual segmentation results for the SCF (+0.1 cm^2^/+0.1%), quadriceps (±0.0 cm^2^/±0.0%), hamstring (+0.6 cm^2^/+2.0%) and adductor (+0.2 cm^2^/+2.2%) CSAs (Fig. 6). Notable systematic deviations between the semi-automated and the manual segmentation methods were observed for the sartorius (−0.1 cm^2^/−4.2%) and IMF CSAs (+4.6 cm^2^/+38.1%) (Fig. 8).

### Inter-observer variability

The inter-observer variability (RMS CV%) for the semi-automated method was 0.9% for the SCF, 1.9% for the IMF, 0.4% for the quadriceps, 0.5% for the hamstrings and 0.9% for the adductors. No statistically significant systematic differences between the results of both observers were noted (paired *t* test), except from the sartorius CSAs with a RMS CV% of 2.1% (*p* = 0.04).

### Longitudinal validation in the intervention study

In the strength training group, both the manual and semi-automated segmentation showed statistically significant reductions in SCF and IMF CSAs and statistically significant gains in CSAs of the quadriceps, hamstrings and adductors, which did not differ significantly between the two techniques (*p* ≥ 0.54, Table 2). A significant increase in the CSA of the sartorius was observed with the manual but not with the semi-automated segmentation technique (Table 2, *p* = 0.10). Except for the sartorius and adductors, the sensitivity to change (SRM) in adipose and muscle tissue of the semi-automated method tended to be greater than the SRM obtained from the manual segmentations (Table 2).

**Table 2:** Baseline values (mean ± SD, cm^2^), longitudinal change (mean ± SD, in %) and sensitivity to change [standardized response mean (SRM)] in thigh muscle and adipose tissue CSAs observed after 12 weeks of lower-limb strength training using manual and semi-automated segmentation

	SCF	IMF	Quadriceps	Hamstrings	Adductors	Sartorius
Manual						
Baseline (cm^2^)	79.5 ± 26.0	10.5 ± 2.2	49.1 ± 6.4	29.4 ± 4.9	10.9 ± 3.3	2.9 ± 0.4
Change (%)	−5.9 ± 10.2	−12.2 ± 17.2	2.7 ± 4.1	3.1 ± 5.5	10.4 ± 12.5	5.3 ± 7.4
SRM	−0.58	−0.71	0.66	0.58	0.83	0.72
*p*	0.035	0.015	0.018	0.036	0.005	0.012
Semi-automated						
Baseline (cm^2^)	79.2 ± 26.6	15.1 ± 2.5	49.2 ± 6.1	30.1 ± 5.0	11.4 ± 3.6	2.8 ± 0.4
Change (%)	−6.0 ± 9.8	−10.4 ± 12.8	2.7 ± 3.4	3.4 ± 5.2	9.3 ± 15.5	0.4 ± 11.0
SRM	−0.61	−0.81	0.80	0.65	0.60	0.04
*p*	0.027	0.005	0.006	0.021	0.030	0.887
Δ (M vs. S-A)						
Change (%)	0.0 (−2.9, 2.8)	1.8 (−7.0, 10.5)	0.0 (−1.4, 1.3)	0.2 (−1.9, 2.3)	−1.1 (−7.9, 5.6)	−4.9 (−10.9, 1.1)
*p*	0.843	0.759	0.896	0.681	0.544	0.103

Δ mean change manual (M) vs. semi-automated (S-A) in % (95% confidence interval), *SRM* standardized response mean, *p p*-value computed using paired *t* test

**Fig. 9:**
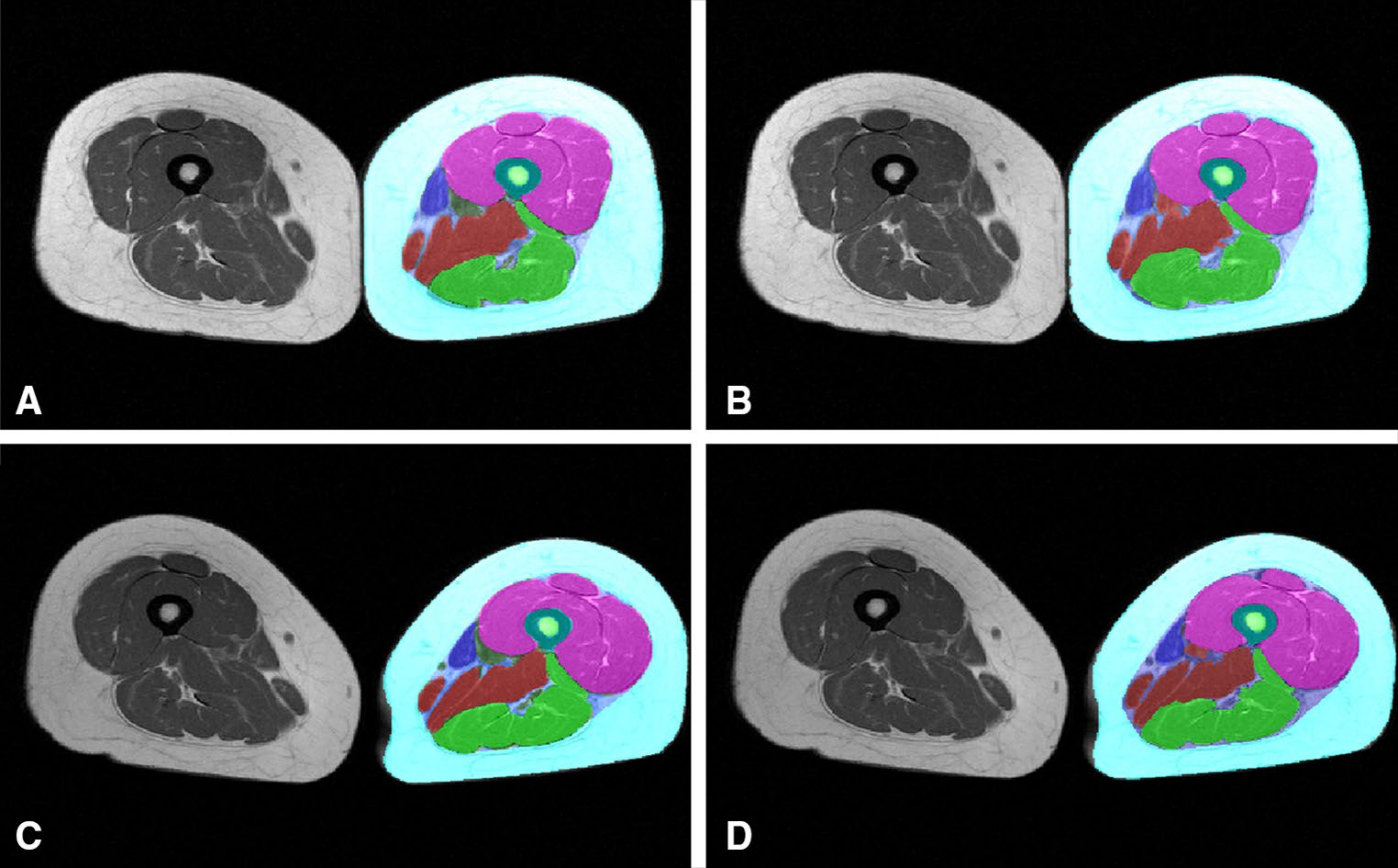
Segmentation results obtained in the endurance training study: **a** manual segmentation at baseline; **b** semi-automated segmentation at baseline; **c** manual segmentation at 12-week follow-up; **d** semi-automated segmentation at 12-week follow-up

In the endurance training intervention group, a significant decrease in the SCF and IMF CSAs and a significant increase in the sartorius CSA were observed with both segmentation methods (Table 3). In contrast, only small, statistically non-significant changes were observed in quadriceps, hamstring and adductor CSAs using both the semi-automated and the manual segmentation method (Table 3). No significant differences were observed when comparing the longitudinal changes between the semi-automated and manual segmentation method (*p* ≥ 0.23, Table 3). In this group, the sensitivity to change tended to be somewhat lower for the semi-automated than for the manual segmentation method (Table 3). Examples of segmentation results obtained in the endurance training study by the semi-automated and manual segmentation method applied at baseline and 12-week follow-up MR images are shown in Fig. 9.

**Table 3:** Baseline values (mean ± SD, cm^2^), longitudinal change (mean ± SD, in %) and sensitivity to change [standardized response mean (SRM)] in thigh muscle and adipose tissue CSAs observed after 12 weeks of lower-limb endurance training using manual and semi-automated segmentation

	SCF	IMF	Quadriceps	Hamstrings	Adductors	Sartorius
Manual						
Baseline (cm^2^)	90.4 ± 40.0	9.6 ± 2.5	47.5 ± 5.1	29.4 ± 4.9	11.0 ± 4.5	3.1 ± 0.6
Change (%)	−6.3 ± 11.1	−14.0 ± 22.1	2.0 ± 6.2	−0.6 ± 5.3	−1.6 ± 31.7	3.5 ± 6.2
SRM	−0.57	−0.63	0.32	−0.11	−0.05	0.57
*p*	0.023	0.013	0.183	0.623	0.823	0.022
Semi-automated						
Baseline (cm^2^)	91.4 ± 41.8	15.0 ± 4.2	47.3 ± 5.3	30.6 ± 3.5	10.8 ± 4.3	2.8 ± 0.5
Change (%)	−4.8 ± 9.3	−15.8 ± 28.3	1.5 ± 5.3	0.4 ± 6.4	−0.9 ± 22.5	8.2 ± 16.3
SRM	−0.51	−0.56	0.27	0.06	−0.04	0.50
*p*	0.040	0.026	0.248	0.813	0.869	0.041
Δ (M vs. S-A)						
Change (%)	1.6 (−2.0, 5.1)	−1.8 (−20.6, 17.1)	−0.5 (−1.5, 0.5)	1.0 (−1.6, 3.5)	0.8 (−10.6, 12.2)	4.7 (−2.8, 12.2)
*p*	0.430	0.539	0.332	0.451	0.935	0.229

Δ mean change manual (M) vs. semi-automated (S-A) in % (95% confidence interval), *SRM* standardized response mean, *p p*-value computed using paired *t* test

## Discussion

The objective of this study was to validate a software solution for the efficient, semi-automated thigh muscle CSA segmentation from MR images that can be applied for the analysis of large longitudinal image repositories such as the OAI. For this purpose, we determined the agreement between semi-automated segmentations and pre-existing manual segmentations using MR images from two different study protocols to ensure that the algorithm can be applied to MR images acquired using an image acquisition protocol different from the one used for training the model. In a second step, we compared the longitudinal changes detected using the semi-automated segmentation technique during a training intervention study with those observed from pre-existing manual segmentations. The results from the current study showed high agreement (DSCs ≥ 0.95) between the semi-automated vs. manual approach for SCF, quadriceps, hamstring and femoral bone segmentations, independent of whether the algorithm was applied to the same type of images on which it has been trained (i.e., the OAI) or to images acquired on a different MR scanner (i.e., training intervention study). The agreement was somewhat less, but still acceptable, for other thigh anatomical structures such as the adductors and sartorius and for the IMF. These results were confirmed by the Bland-Altman analysis: the proposed semi-automated algorithm showed good agreement with the manual segmentations, except for systematic differences observed in sartorius and IMF CSAs. The inter-observer variability was somewhat lower than previously observed using manual thigh CSA segmentations of the same set of thigh MRIs [40]. When applied to the data from the training intervention study, the proposed semi-automated algorithm detected longitudinal changes in muscle and adipose tissue with similar sensitivity to change (SRMs) as previously reported by manual segmentation, but with substantially less time needed for the analysis (3–6 vs. 60–90 min) depending on the reader and the image quality.

The agreement of the semi-automated segmentation results with manual reference segmentations (DSC) for the quadriceps, SCF and IMF (0.70–0.97) was in the same range or higher than the agreement observed for thigh muscle CSAs in previous studies. Prescott et al. [23] used a level set method initialized by anatomically attached templates (separately for men and women) for the semi-automated segmentation of individual quadriceps heads in 53 subjects from the OAI and reported DSCs between 0.69 ± 0.16 (rectus femoris) and 0.82 ± 0.08 (vastus lateralis) [23]. Trotter et al. used a fully-automated multi-atlas and a semi-automated single-atlas method for segmenting individual quadriceps heads and reported a mean DSC of 0.87 ± 0.11 for the fully automated multi-atlas method and DSCs of >0.90 for the semi-automated single-atlas method [27]. Baudin et al. used a “random walks graph”-based algorithm, initiated by a statistical shape atlas and reported a mean DSC of 0.86 ± 0.07 for individual thigh muscle heads [24]. Andrews et al. employed a probabilistic shape representation for the segmentation of the thigh muscle heads and reported a mean DSC of 0.81 ± 0.07 [28]. Using a voxel classifier-based technology combined with morphological operations for the segmentation of thigh muscles and adipose tissue, Yang et al. [26] reported DSCs of 0.96 ± 0.03 for the SCF, 0.80 ± 0.03 for the IMF and 0.97 ± 0.01 for the combined thigh muscles (quadriceps, hamstrings, adductors and sartorius) when the algorithm was applied to four contrast Dixon MR images and somewhat lower DSCs when the algorithm was applied to fat and water suppressed (SCF 0.94 ± 0.04, IMF 0.68 ± 0.10 and combined thigh muscle 0.96 ± 0.03) or unsuppressed (SCF 0.80 ± 0.10, IMF 0.37 ± 0.13 and combined thigh muscle 0.73 ± 0.21) MR images only. Karlsson et al. used a multi-atlas segmentation approach to automatically segment the lean muscle tissue from whole body intensity corrected water-fat separated MRIs and reported a true positive volume fraction of 0.93 ± 0.01 to 0.93 ± 0.03 for the automated thigh segmentation [29]. These approaches are, however, not directly applicable to data from the OAI, where only T1-weighted spin-echo images of the thigh were acquired [26, 29]. Also, it is important to note that the DSC depends on both the size of the object and the complexity of the object boundaries, because the segmentation errors occurring at the object boundaries are related to the voxels inside the object, which are typically in agreement between methods. The results from the current study, in which the entire quadriceps muscle was segmented semi-automatically, and the results from the studies focusing on the (semi-) automated segmentation of the individual quadriceps heads [23–25, 27] or the combination of all thigh muscles together [26] should therefore be compared with caution. However, previous approaches were not validated for the assessment of longitudinal changes in thigh tissue composition. We overcome this limitation by measuring longitudinal change in thigh composition and were able show a similar sensitivity to change as manual segmentation. The ability to capture even rather small longitudinal changes and the high agreement achieved between the semi-automated method and manual segmentations in our current study are promising for the application of the semi-automated method in future studies, in particular for the analysis of SCF, quadriceps and hamstring CSAs, which are the primary focus in knee OA [3, 13] or in training intervention studies [41]. Future studies using this technique should, however, employ a central quality control by an expert reader to ensure accurate and consistent segmentation results.

A limitation of the presented semi-automated segmentation method is that it is currently limited to the segmentation of a particular anatomical location and does not yet allow for volumetric analysis. The approach may also not be appropriate for conditions that affect the skeletal muscles inhomogeneously, whereas in KOA, muscle CSAs are used as surrogate markers. Previous analyses showed that single-slice quadriceps and hamstring CSAs located at 33% of the femoral length (distal to proximal) were sensitive to change in KOA [12], correlated with 3D quadriceps and hamstring muscle volume [42], and that the effect size of detecting between-limb differences in participants with unilateral pain was similar for one vs. several MRI slices [11]. Also, the OAI thigh MR images do not cover the entire thigh, and the method used in the current study was tailored to the analysis of the slice located at 33% of the femoral length (distal to proximal), which can be consistently selected throughout almost all participants with the thigh MRI protocol used by the OAI [11, 17]. However, the current approach should in principle also work in the same way at other levels of the thigh if sufficient numbers of training data sets are available. An extension of the semi-automated segmentation method presented here to the analysis of a greater number of slices or the entire 3D volume of the thigh seems therefore feasible. Whether such a 3D segmentation would require training based on suitable data sets or whether the results from the single-slice analysis can be used for initializing the segmentation of adjacent slices remains to be determined. Another potential limitation of the current study is that the validation of the approach did not include the selection of the slice located at 33% of the femoral length (distal to proximal), because this had already been done when performing the manual reference segmentations. Selecting the target slice can, however, be easily performed, in particular if the entire femur is depicted in the 3D MRI data set like it was in the case in the training study. Another limitation of our approach is that the DSC observed for the IMF and the adductors was relatively low, and the Bland-Altman plots showed that the semi-automated approach systematically overestimated the IMF. This was caused by the thresholding method that is used to separate the adductors and the inter-muscular fat from other inter-muscular tissue (e.g., veins, arteries). We have explored the use of *k*-means clustering and Otsu’s thresholding method for this purpose, but these methods did not provide better results than the thresholding method used in this study. Yet, the DSC observed for IMF in the current study is in the same range as the DSC observed for IMF in the study of Yang et al. [26]. This indicates that the IMF is a quite complex structure, and a thorough quality control and manual corrections will currently be required to reliably measure the CSAs of the adductors and the IMF. However, since the observed effect is systematic and similarly applies to baseline and follow-up data, measuring longitudinal change in the IMF (i.e., during training intervention or weight loss) should not be strongly affected.

A strength of the current study is that the agreement between the semi-automated and the manual reference segmentations was not only assessed on MR images acquired using the same imaging protocol and equipment but also on MR images acquired using a different imaging protocol on a scanner from a different manufacturer. The agreement obtained for MR images from the OAI was comparable to the agreement observed for MR images from the training intervention study, although the semi-automated segmentation method was only trained using MR images from the OAI. More importantly, the longitudinal increase in muscle CSAs and the reduction in adipose tissue CSAs previously reported using manual segmentations from the training intervention study [19] were reproduced with a comparable sensitivity to change. The longitudinal changes detected by the semi-automated segmentation approach in the training intervention study were in the same range as those reported from other longitudinal studies [14, 18], and, despite the somewhat lower DSC agreement, the algorithm also was able to detect a longitudinal reduction in IMF CSAs, which have been reported to be of particular importance in knee OA [14, 18].

The DSC obtained from the twofold cross-validation experiment was highly consistent between the two times ten repetitions, and it was also only slightly lower than the DSC obtained when applying the full ASM model in combination with the ACM post-processing. This indicates that a model smaller than the one used in this study might be sufficient for segmenting thigh CSAs from MRI. To explore the relationship between the size of the ASM model and the segmentation performance, another experiment was performed in which the size of the ASM model was iteratively increased in steps of five. The results from this experiment showed that, as expected, increasing the size of the training data set led to improved segmentation performance. However, this effect became smaller when the size of the model was greater than *n* = 50 training data sets. Beyond this size, the segmentation performance still improved and the results became more consistent, but the improvement was relatively small. The segmentation of thigh CSAs from MRI therefore appears to be feasible when using ASMs smaller than the one used in the current study, although the results will most likely be somewhat inferior to the results obtained when using the full model. Changes in muscle CSAs are less variable and more robust than changes in the clinical measurement of muscle strength [12], but when done manually, a lot of effort and time are typically required for segmentation. The semi-automated approach proposed here, however, will permit the investigation of thigh muscle and adipose tissue CSAs in very large cohorts in a realistic time period. Given that the OAI image repository contains publicly available axial MR images of the thigh from almost 5000 participants at baseline, 2- and 4-year follow-up, the technique presented here is particularly useful in exploring the role of thigh muscle loss and longitudinal adipose tissue changes in the development and progression of knee OA.

## Conclusions

Using a well-trained active shape model (ASM) combined with an active contour model (ACM), we have shown high cross-sectional agreement between semi-automated and manual segmentation methods for thigh muscle and adipose tissue CSAs. Importantly, the time-efficient semi-automated algorithm detected longitudinal training effects on muscle and adipose tissue of the thigh with a similar sensitivity to change as manual thigh CSA segmentations. This novel approach can be used to evaluate thigh CSA morphology from large data sets with much less time and with equivalent accuracy as manual segmentation.

## Electronic supplementary material

Below is the link to the electronic supplementary material.

Supplementary material 1 (DOCX 16 kb)
