# Supplementary material for: Validation of an active shape model-based semi-automated segmentation algorithm for the analysis of thigh muscle and adipose tissue cross-sectional areas
Source: MAGMA. 2017 Apr 28;30(5):489–503. doi: 10.1007/s10334-017-0622-3 (PMC5608793; doi:10.1007/s10334-017-0622-3)
Supplement: Supplementary file 1 — Supplementary material 1 (DOCX 16 kb) [file 10334_2017_622_MOESM1_ESM.docx]

Supplemental table 1: Average dice similarity coefficients (DSC; agreement between manual and semi-automated segmentation; mean±SD) in n=10 rounds of 2-fold cross-validation partitioning the 113 training data sets randomly in two subsets (n = 56 and n = 57). The model was trained using the data from subset 1 and tested on the data from subset 2 (and vice versa).

|  | SCF | | Quadriceps | | Hamstrings | | Sartorius | | Femur | | Medulla | |
| --- | --- | --- | --- | --- | --- | --- | --- | --- | --- | --- | --- | --- |
| n | Subset 1 | Subset 2 | Subset 1 | Subset 2 | Subset 1 | Subset 2 | Subset 1 | Subset 2 | Subset 1 | Subset 2 | Subset 1 | Subset 2 |
| 1 | 0.90±0.05 | 0.91±0.04 | 0.93±0.03 | 0.93±0.02 | 0.88±0.05 | 0.88±0.05 | 0.77±0.11 | 0.74±0.13 | 0.92±0.02 | 0.92±0.02 | 0.87±0.05 | 0.88±0.05 |
|  |  |  |  |  |  |  |  |  |  |  |  |  |
| 2 | 0.90±0.05 | 0.91±0.05 | 0.94±0.02 | 0.94±0.03 | 0.88±0.05 | 0.88±0.04 | 0.78±0.12 | 0.78±0.14 | 0.92±0.03 | 0.91±0.03 | 0.88±0.05 | 0.86±0.06 |
|  |  |  |  |  |  |  |  |  |  |  |  |  |
| 3 | 0.91±0.05 | 0.90±0.05 | 0.93±0.03 | 0.94±0.02 | 0.87±0.05 | 0.89±0.05 | 0.79±0.12 | 0.78±0.12 | 0.91±0.03 | 0.92±0.03 | 0.85±0.06 | 0.88±0.05 |
|  |  |  |  |  |  |  |  |  |  |  |  |  |
| 4 | 0.91±0.05 | 0.91±0.04 | 0.94±0.02 | 0.94±0.03 | 0.88±0.05 | 0.88±0.05 | 0.80±0.11 | 0.77±0.14 | 0.91±0.03 | 0.92±0.02 | 0.87±0.06 | 0.86±0.05 |
|  |  |  |  |  |  |  |  |  |  |  |  |  |
| 5 | 0.91±0.05 | 0.91±0.05 | 0.93±0.03 | 0.94±0.02 | 0.88±0.04 | 0.88±0.05 | 0.78±0.11 | 0.79±0.14 | 0.92±0.03 | 0.92±0.03 | 0.86±0.06 | 0.87±0.06 |
|  |  |  |  |  |  |  |  |  |  |  |  |  |
| 6 | 0.91±0.05 | 0.91±0.04 | 0.94±0.02 | 0.93±0.03 | 0.89±0.04 | 0.88±0.05 | 0.79±0.12 | 0.76±0.13 | 0.92±0.03 | 0.91±0.03 | 0.86±0.06 | 0.87±0.06 |
|  |  |  |  |  |  |  |  |  |  |  |  |  |
| 7 | 0.90±0.05 | 0.91±0.04 | 0.94±0.02 | 0.94±0.02 | 0.88±0.05 | 0.88±0.05 | 0.78±0.11 | 0.78±0.13 | 0.92±0.03 | 0.91±0.03 | 0.87±0.05 | 0.86±0.06 |
|  |  |  |  |  |  |  |  |  |  |  |  |  |
| 8 | 0.90±0.04 | 0.92±0.05 | 0.94±0.02 | 0.94±0.03 | 0.88±0.04 | 0.88±0.05 | 0.77±0.12 | 0.79±0.12 | 0.92±0.03 | 0.91±0.03 | 0.87±0.05 | 0.86±0.06 |
|  |  |  |  |  |  |  |  |  |  |  |  |  |
| 9 | 0.91±0.05 | 0.91±0.05 | 0.94±0.02 | 0.93±0.03 | 0.89±0.04 | 0.87±0.05 | 0.78±0.13 | 0.79±0.12 | 0.92±0.03 | 0.91±0.03 | 0.88±0.05 | 0.85±0.06 |
|  |  |  |  |  |  |  |  |  |  |  |  |  |
| 10 | 0.90±0.05 | 0.90±0.05 | 0.94±0.02 | 0.94±0.02 | 0.88±0.05 | 0.88±0.05 | 0.77±0.14 | 0.77±0.14 | 0.92±0.03 | 0.92±0.03 | 0.88±0.05 | 0.88±0.05 |
